# A single-institution study of stereotactic body radiotherapy for patients with unresectable visceral pulmonary or hepatic oligometastases

**DOI:** 10.1186/1748-717X-7-164

**Published:** 2012-09-27

**Authors:** Ingrid Fumagalli, Jean-Emmanuel Bibault, Sylvain Dewas, Andrew Kramar, Xavier Mirabel, Bernard Prevost, Thomas Lacornerie, Hajer Jerraya, Eric Lartigau

**Affiliations:** 1 Radiation Therapy Department, Oscar Lambret Comprehensive Center Lille, 3 rue Combemale, Lille cedex, 59020, France; 2 Biostatistics Department, Oscar Lambret Comprehensive Cancer Center, Lille, France; 3 Medical Imaging Department, Oscar Lambret Comprehensive Cancer Center, Lille, France

**Keywords:** SBRT, Liver metastasis, Lung metastasis, Oligometastases, CyberKnife

## Abstract

**Purpose:**

The purpose of this study is to evaluate the feasibility, efficacy and toxicity of SBRT for treatment of unresectable hepatic or lung metastases regardless of their primary tumor site for patients who received prior systemic chemotherapy.

**Methods and materials:**

Between July 2007 and June 2010, 90 patients were treated with the CyberKnife® SBRT system for hepatic or pulmonary metastatic lesions. Medical records were retrospectively reviewed. The endpoints of this study were local control, overall survival (OS), disease-free survival (DFS), local relapse free-survival (LRFS), and treatment toxicity.

**Results:**

A total of 113 liver and 26 lung metastatic lesions in 52 men (58%) and 38 women (42%) were treated. Median follow-up was 17 months. Median age at treatment was 65 years (range, 23–84 years). Primary cancers were 63 GI, three lung, eight breast, four melanoma, three neuro-endocrine tumors, and three sarcomas. Median diameter of the lesions was 28 mm (range, 7–110 mm) for liver and 12.5 mm (range, 5–63.5 mm) for lung. Local control rates at 1 and 2 years were 84.5% and 66.1%, respectively. Two-year overall survival rate was 70% (95% CI: 55–81%). The 1 and 2-year disease-free survival rates were 27% (95% CI: 18–37%) and 10% (95% CI: 4–20%), respectively. Median duration of disease-free survival was 6.7 months (95% CI: 5.1–9.5 months). Observed toxicities included grade 1–3 acute toxicities. One grade 3 and no grade 4 toxicity were reported.

**Conclusion:**

High-dose SBRT for metastatic lesions is both feasible and effective with high local control rates. Overall survival is comparable with other available techniques. Treatment is well tolerated with low toxicity rates. It could represent an interesting treatment option for oligometastatic patients not amenable to surgery, even when patients had been pre-treated with chemotherapy.

**Summary:**

Stereotactic body radiotherapy (SBRT) has previously been successfully used in the treatment of metastatic lesions. It could be considered as a curative option for oligometastatic patients. This retrospective study involved 90 patients, designed to test potential effectiveness of SBRT in the treatment of oligometastases irrespective of primary. Results suggest SBRT could be an effective treatment extending patients’ life span. This treatment appears to be more effective when used prior to multiple systemic treatment regimens.

## Introduction

Over the past decade, stereotactic body radiation therapy (SBRT) has emerged as an alternative treatment option for patients with liver [1,2] and lung [3,4] lesions. The term "oligometastases" was first described by Hellman and Weichselbaum [5] in 1995 as a less-advanced state of metastatic disease amenable to and potentially curable with local therapy. The term "oligometastases" is most often used to describe five or fewer metastatic lesions [6]. Often, this pathology has a slow rate of progression, justifying focal treatments.

Main therapeutic options for oligometastases remain surgery or radiofrequency. Due to its high level of precision which allows hypofractionnation, SBRT has gained interest as a curative treatment option for inoperable patients with lung cancer, liver tumors, or oligometastases. The concept of local treatment for oligometastatic lesions exists since only a few years [7-9] Researchers have reported encouraging data for liver metastases treated with SBRT. Katz et al. [10] have reported a local control rate of 76% and 57% at 10 and 20 months, respectively, with 69 patients. For lung metastases, Rusthoven et al. have reported local control rates of 100% and 96% at 1 and 2 years, respectively [11]. SBRT can be delivered without tumor tracking using a stereotactic frame or the breath-hold technique, or most recently, with a real-time tumor tracking system, which is available with the CyberKnife® System (Accuray Incorporated, Sunnyvale, CA). Most studies have reported results from treatments delivered without tracking [12]. With real-time tracking, patients can breathe freely during treatment sessions.

Milano reported a study of 121 patients with oligometastases from any site analyzing survival and tumor control with encouraging results [6]. Bone metastases appeared to be associated with a fourfold reduced hazard of death. To our knowledge, no previous study has been reported with this technique of SBRT for a pooled analysis of lung and liver metastases.

We report a retrospective study of 90 patients with 139 visceral oligometastases (lung or liver), regardless of the primary analyzing overall survival, local control and relapse free survival.

### Patients and methods

#### Patients

Patients treated between July 2007 and June 2010 were included and medical records were retrospectively reviewed. Patients with oligometastases (1 to 5 lesions) from any primary site and not amenable to surgery, due most of the time to anesthesia were eligible. Other inclusion criteria were a Performance Status >3 (WHO scale), number of hepatic or lung lesions <5, and lesion size <100 mm for hepatic metastases and <70 mm for lung lesions. Clinical data on 90 patients presenting with 113 hepatic and 26 lung metastases were collected and analyzed. Patients and tumor characteristics are presented in Table 1. Treatment response was assessed using the RECIST 1.1 criteria. Toxicity was evaluated with the CTCAE v4.0. Written informed consent was obtained from the patient for publication of this report in accordance with the guidelines of the French National Cancer Institute (Institut National du Cancer) required when assessing the efficacy and toxicity of a novel therapy,

**Table 1:** Patient characteristics

**Patients**	**Total (n = 90)**	**%**
Gender: Female	38	42%
Male	52	58%
Age at diagnostic, median (range):	62 years	(22–82)
Age at SBRT, median (range):	65 years	(23–84)
Primary sites: Digestive	63	70%
Lung	3	3.3%
Breast	8	8.9%
Melanoma	4	4.4%
Neuro-endocrine	3	3.3%
Sarcoma	3	3.3%
Other	6	6.7%
Adenocarcinoma Histology: Hepatic (n = 75)	61	81%
Lung (n = 15)	7	47%
Primary for Adenocarcinoma histology	68	
Colorectal	57	84%
Breast	8	12%
Gastric	1	1.5%
Lung	1	1.5%
Kidney	1	1.5%
Primary for squamous cell histology :	7	
Esophagus	5	71%
Lung	2	29%
Other histologies :	15	17%
Lesions : Lung	15	17%
Hepatic	75	83%
Number of lesions treated by patient :		
1	64	71%
2	19	21%
3	4	4%
4	3	3%
Timing of Metastases:		
At initial diagnostic	43	49%
Within one year	20	23%
More than 1 year	25	28%
Time from diagnosis of metastasis to SBRT treatment, median (range)	25.6 months	(1.2 – 93.6)
Previous local treatment :		
Hepatic (n = 75)	53	71%
Lung (n = 15)	0	0%
Prior chemotherapy:		91%
Hepatic lesions (n = 75)	70	93%
Lung lesions (n = 15)	12	92%
Prior progressive disease with Chemo:		
No	67	82%
Yes	15	18%
More than 3 chemotherapy regimens before SBRT :		26,6%
Hepatic lesions (n = 75)	21	28%
Lung lesions (n = 15)	3	20%

## Methods

SBRT was delivered with Cyberknife, an integrated image-guided frameless stereotactic radiation therapy system. It is comprised of a robotic arm coupled with a compact 6-MV X-Ray linear accelerator and a real-time imaging system. Different collimators with diameters ranging from 5–60 mm are used with the linac. Two X-Ray tubes are positioned in the treatment room at a 45° angle. Images created are registered to the treatment-planning console. This system detects a difference of 0.06 mm in translation and rotation. For liver metastases, all patients were treated with the Synchrony® Respiratory Tracking System. Four gold fiducial markers were inserted around the lesion 2 weeks before the treatment planning. For lung metastases, patients could be treated with either Xsight® Lung Tracking System (Accuray) or, using an internal target volume (ITV), with Xsight® Spine Tracking System (Accuray). These tracking methods did not require the implantation of fiducial markers. For the planning computed tomography (CT) study, patients were immobilized in an external vacuum-type body mold. An abdominal belt could be used for liver lesions in order to decrease respiration amplitudes.

A 4D CT-scan was performed for patients with liver or lung metastases, respectively, with a 1-mm slice thickness and reconstruction in 3-mm slices. For the lung patients tracked with Xsight Spine, ITV was defined using the data from the 4D CT-Scan performed for treatment planning. For patients treated with tumor tracking, GTV was contoured. GTV or ITV was expanded by 5 mm to create the clinical tumor volume (CTV). CTV was expanded by 3 mm to obtain the planning target volume (PTV). SBRT planning was performed with Accuray’s Multiplan® treatment planning software. Dose was prescribed to the 80% isodose line and delivered to the target volume over a mean duration of 8 days (range, 3–22 days). The median fractionnation scheme was three fractions of 15 Gy (range, 9–20 Gy).

Critical organ dose constraints are detailed in Table 2.

**Table 2:** Critical organ constraints

		**Constraints**
Liver metastases	Normal liver	V21 < 33%
		V15 < 50%
	stomach	V21 < 5 cm^3^
	Spinal cord	maximal dose < 22 Gy
	Kidney	V 15 < 33%
Lung metastases	normal lung (= volume of right + left lung – PTV)	V5 < 50%
		V10 < 30%
	One lung	V 20 < 20%
	Heart	V24 < 15 cm^3^
		Dmax < 30 Gy
	trachea and principal bronchi	V 15 < 4 cm^3^
		V 20 < 1 cm^3^
		Dmax < 30 Gy
	esophagus	V 21 < 5 cm^3^
		Dmax < 25 Gy

### Follow-up

All patients were seen every 3 months with a new CT-Scan. Treatment response was evaluated every 3 months using the RECIST 1.1 criteria: complete response (CR) defined as complete disappearance of tumor or only fibrosis in the image, partial response (PR) defined as tumor shrinkage of at least 30%, stable disease (SD) defined as no radiologically measurable difference and progressive disease (PD) defined as a tumor increase of at least 20%. Local control was defined as CR, PR, or SD during follow-up based on imaging. Acute toxicity was evaluated using CTCAE v4.

### Statistics

Descriptive statistics were used for categorical variables (frequency and percentage) and continuous variables (median and range). The following variables were analyzed: age, gender, primary site, histology, previous local or systemic treatment, time from initial diagnosis to first metastasis, time from the diagnosis of the first metastasis to SBRT, total dose received, number of fractions, dose per fraction, size of the target lesions, and treatment time. All time to event endpoints were calculated from the initiation of treatment. Overall survival included death from any cause. Disease-free survival counted events as a progression of an existing target lesion, appearance of new lesions within the same organ (liver or lung), distant relapse, or death due to cancer, whichever occurred first. Local failure included events involving pre-existing treated lesions only. Survival rates were estimated by the Kaplan-Meier method and groups were compared with the logrank test. P values smaller than 0.05 were considered statistically significant.

## Results

### Patient characteristics

Ninety patients were treated (52 male and 38 female). Median age at diagnosis was 62 (range, 22–82) and median age at the time of SBRT was 65 (range 23–84). Seventy-one percent of patients had only one metastatic lesion. No patient had both liver and lung lesions. Histologies of the metastatic lesions were: 68 adenocarcinomas including 57 colorectal tumors, eight breast tumors, one stomach cancer, one lung, and one kidney cancer case; seven squamous-cell carcinomas from esophagus and lung; and 15 other histologies [four melanomas, three neuro-endocrine tumors, three sarcomas, one GIST (gastro-intestinal stromal tumor), one papillary thyroid tumor, one pneumoblastoma, one adenoid cystic cancer, two small-cell lung cancers]. Forty-nine percent of the patients had metastases at the time of initial diagnosis. Median time from diagnosis of metastases to start of CyberKnife treatment was 25.6 months (range, 1.2–93.6 months). Eighty-five percent of patients had received prior chemotherapy; 93% and 92% of liver and lung patients, respectively. Twenty-seven percent of patients treated with SBRT had received more than three cycles of chemotherapy prior to CyberKnife, 28% and 21% of liver and lung patients, respectively. No patients had received prior radiotherapy.( Table 1)

There were 113 hepatic and 26 lung metastatic lesions (Table 3). Median lesion diameter was 28 mm (range, 7–100) for liver and 12.5 mm (range, 5–63.5) for lung. CR rate was 52%, PR 18%, SD 8%, and PD 22%. Except for one patient, none of the patients with lung metastases had any previous local treatment compared to the 53/75 (71%) patients with 68/113 (69%) hepatic metastases who were previously treated locally with surgery to the treated lesion (2%), surgery to other lesions (44%), chemo-embolization (3%), or radio frequency ablation (12%).

**Table 3:** Characteristics and response of metastatic lesions

**Lesions**	**Total (n = 139)**	**%**
Number of lesions:		
Hepatic metastases	113	81%
Lung metastases	26	19%
Lesion diameter, median (range):		
Liver (n = 111)	28 mm	(range 7–100)
Lung (n = 26)	12.5 mm	(range 5–63.5)
Previous local treatment to hepatic lesions	68	60%
Local hepatic treatment:		
Surgery of same lesion	2	2%
Surgery of other lesions	49	44%
Chemo-embolisation	3	3%
Radio-frequency	14	12%
Response (RECIST)		
Hepatic metastases		
CR	56	51%
PR	22	20%
SD	10	9%
PD	22	20%
Response		
Lung metastases		
CR	14	58%
PR	2	8%
SD	1	4%
PD	7	29%
Response		
All lesions		
CR	70	52%
PR	24	18%
SD	11	9%
PD	29	20%

### Treatment characteristics

Fifty eight patients were treated with three fractions of 9 Gy (1.1%), 10 Gy (2,2%); 12 Gy (3.3%), 13 Gy (2.2%), 15 Gy (53.3%), or 20 Gy (2.2%) times (Table 4). The 20 Gy per fraction regimen was only administered for lung metastases. Thirty patients (35.6%), were treated with 4 fractions of 10 Gy. One patient was treated with six fractions of 6 Gy for a total dose of 36 Gy to a lung metastasis because of the central localization of the lesion and one patient was treated with 6 fractions of 9 Gy for a liver metastasis for a total dose of 54 Gy due to the duodenum proximity. All patients received a total dose of 27–60 Gy (median dose, 45 Gy). Median treatment time was 8 days (range, 3–22) ( Table 4). Treatment was delivered in two weeks, with a session being performed every 2 or 3 days.

**Table 4:** Cyberknife treatment characteristics (by patients)

**Patients**	**Hepatic (n = 75)**	**Lung (n = 15)**	**All Patients (n = 90)**
Dose per fractions, n (%)			
6	0	1	1 (1,1%)
9	2	0	2 (2,2%°
10	31 (41%)	1	32 (35,6%)
12	3	0	3 (3,3%)
13	2	0	2 (2,2%)
15	37 (49%)	11 (73%)	48 (53,3%)
20	0	2	2 (2,2%)
Total dose, n (%)			
27	1	0	1,1%
30	2	0	2,2%
36	3	1	4,4%
39	2	0	2,2%
40	29 (39%)	1	30 (33,3%)
45	37 (49%)	11 (73%)	48 (53,3%)
54	1	0	1,1%
60	0	2	2,2%
Treatment duration, median (range)	8 days(3 – 22)		

### Treatment efficacy

**Figure 1:**
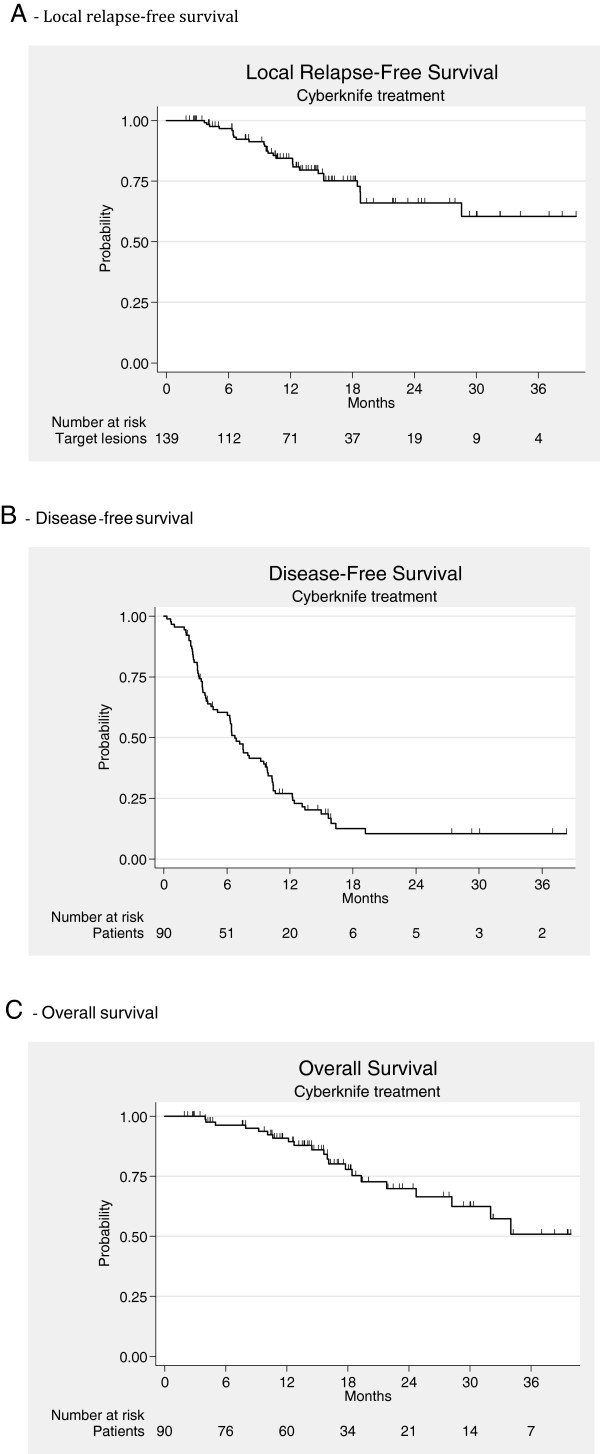
**Kaplan-Meier Survival curves. A.** Local relapse-free survival. **B**. Disease-free survival. **C**. Overall survival.

Median follow up was 17 months (95%CI 14-21). The overall response rate was complete for 52% of the lesions, partial for 20%, stable for 9%, and progressive for 20% of the lesions. Results were similar for hepatic and lung lesions. At last follow-up, 21 patients had died and 17 patients were disease-free. The local control rate at 1 and 2 years were 84.5% and 66.1%, respectively (Figure 1A). The 1 and 2-year disease-free survival rates were 27% (95% CI: 18–37%) and 10% (95% CI: 4–20%), respectively (Figure 1B). Median duration of disease-free survival was 6.7 months (95% CI: 5.1–9.5 months). The 2-year overall survival rate was 70% (95% CI: 55–81%) (Figure 1C).

### Toxicity

Side effects were generally mild. Observed toxicites ranged from grade 1 to 2 in the CTCAE v.4. Toxicity for patients treated for hepatic lesions consisted mainly of digestive adverse events : we recorded 19 nausea (17%), 10 vomiting (9%), three gastritis (2,5%), three anorexia (2,5%), 17 hepatic pain (15%) and 9 asthenia. (8%) Toxicity for patients treated for lung lesions was lower, consisting mainly of asthenia (5 = 19%) radiation pneumonitis (5 = 19%) and pleural effusion (3 = 11,5%). We encountered two grade 3 toxicities : one gastritis in a patient with a hepatic lesion and one epidermitis in a patient treated for two different hepatic lesions.

### Univariate analysis

Regarding local control, neither tumor size nor dose was found to be statistically significant. Adenocarcinoma appeared to present a higher risk of failure than other histologies: HR = 2.74 (95% CI: 0.95–7.88, p = 0.036). Control local rate was 82% for adenocarcinoma compared to 89% for other histologies at 1 year and 59% at 2 years compared to 80% for other histologies. Regarding disease-free survival, patients with lung lesions were at a lower risk of failure than patients with hepatic lesions: HR = 0.47 (95% CI: 0.23–0.95, p = 0.02). Patients with a history of receiving chemotherapy were at higher risk of failure compared to those without: HR = 4.51 (95% CI: 1.10–18.47, p = 0.007) (Table 5). Other factors such as progressive disease following earlier chemotherapy, more than three previous chemotherapy regimens, and time between diagnosis of the first metastasis and treatment by SBRT were not found to be prognostic for disease-free survival. As far as local control is concerned, female patients had a tendency for lower risk of local failure: HR = 0.48 (95% CI: 0.21–1.10, p = 0.07). No factor had any impact on overall survival.

**Table 5:** Predictive factors for disease free survival

	**DFS**	
**Patients**	HR : (95% CI)	(p)
Gender: Female	1.17: (0.74 – 1.86)	0.51
Age at SBRT 65+	0.72: (0.46 – 1.15)	0.17
Adenocarcinoma Histology:	1.28 : (0.75 – 2.19)	0.36
**Lesions : pulmonary**	**0.47 : (0.23 – 0.95)**	**0.02**
Number of lesions : 3+	1.17 : (0.70 – 1.98)	0.55
Number of hepatic lesions : 3+	1.40 : (0.79 – 2.48)	0.25
Timing of Metastases: < one year	0.67: (0.37 - 1.22)	0.19
More than 1 year	1.04: (0.60 – 1.79)	0.90
**Prior chemotherapy**	**4.51: (1.10 – 18.47)**	**0.007**
3+ chemotherapies before SBRT :	1.20: (0.63 – 2.25)	0.59
Prior PD with previous CT	1.31: (0.74 – 2.32)	0.37

## Discussion

### The concept of oligometastasis

Oligometastasis is an emerging paradigm in oncology. It is described as a distant extension of a primary cancer with an isolated site or less than five sites of metastases [6]. Oligometastases could be seen as clinical manifestations of a systemic disease requiring systemic treatment. But they can also be considered as consequences of a slow, controllable disease progression, curable with local treatments. Local treatments include surgery, radiofrequency, conformal or stereotactic body radiation therapy. The use of SBRT has seen rapid growth as a noninvasive treatment modality and provides a new treatment alternative for inoperable or elderly patients. Salama and al. [13] recently published a dose escalation trial in patients with 1 to 5 metastases in SBRT. Maximal dose was 48 Gy but the maximal dose tolerated was not reached. 2 year overall survival rate was 56.7% and progression free survival rate were 33.3% and 22% at one and two years respectively. Our progression-free survival rate were lower but we focused our study on patients with visceral metastasis, which might explain this difference. Indeed in the study published by Salama et al., 13.3% of patients had only bone metastases, which could be a factor of better prognosis, as Milano et al. have shown [6].

### Treatment efficacy and predictive factors

Several studies have been published on different SBRT methods. Most of them report about primary and secondary lesions without distinction [12,14]. Rusthoven et al. have reported 47 patients with 63 hepatic lesions. Among the patients, 69% had received at least one prior systemic therapy regimen for metastatic disease (zero to five regimens) and 45% had extrahepatic disease at study entry. Actuarial in-field local control rates at one and two years after SBRT were 95% and 92%, respectively. These rates are better than those reported in our study. This could be explained by the fact that the Rusthoven study contained only liver patients and 31% of them had not received any chemotherapy before SBRT. There are also few data on lung metastases. Studies report local control rates from 72–100% at 1 year and from 78–96% at 2 years [11,14-16].

Our study evaluated feasibility of SBRT on hepatic and pulmonary oligometastatic lesions from a multitute of primaries. Local control rates at 1 and 2 years were, respectively, 84.5% and 66.1%. These results are similar to other SBRT reports on liver metastases in which 1-year local control rates ranged from 71–100% and 2-year rates from 30–92% [1,17,18]. In our cohort, 49% of the patients had metastases at initial diagnosis. This fact may have affected our results because of the prognosis of such disease that is often considered more aggressive. Adenocarcinoma displayed worse prognosis than other histologies: HR = 2.74 (95% CI: 0.95–7.88), p =0.036. This could suggest that a more potent dosing regimen should be employed when treating metastases with adenocarcinoma pathology. Indeed, dose has not been shown as a predictive factor and treatment was well tolerated. This is probably also related to the localisation of the treated lesions. Majority of adenocarcinoma metastases were hepatic lesions as opposed to being lung lesions. These results were based on a sub-group analysis and care needs to be taken in the interpretation.

Regarding disease-free-survival, patients with lung lesions were at lower risk of failure than those with hepatic lesions (HR = 0.47, 95% CI: 0.23–0.95, p = 0.02). As there is no previous study comparing lung and hepatic metastases treated with SBRT, this result appears to be a new finding to take into account when considering SBRT as a treatment option. However, number of lung lesions was lower than number of liver lesions Furthermore, the total dose and fractionnation were different between lung and hepatic tumors. 11/15 patients with lung tumors received 15 Gy x 3 and 2/15 received 20 Gy x 3 while only 37/75 patients with liver tumors received 15 Gy x 3 and all the rest received lower doses. Another explanation could be that the lesions' median diameter for lung metastases was 12.5 mm versus 28 mm for hepatic lesion. Our univariate analysis did not find a difference in local control based on the primary tumor site, suggesting that SBRT could be used to treat oligometastases from any primary. Still, according to other studies, the role of the primary tumor site in the case of liver metastases remains unclear [18]. Lee and al. didn't find a difference in overall survival between colorectal, breast and other primaries of metastases treated (respectively 63% (95% CI, 44% to 78%), 79% (95% CI, 36% to 94%), and 38% (95% CI, 14% to 62%).

Dose, within dose range used in this study, did not appear to be a factor in disease-free or overall survival. Although, this result has been confirmed in other studies. McCammon *et al.* and al [12] found that increased dose and smaller gross tumor volume were significant predictors of higher local control rates. Their patients treated with 54 Gy or more had a 3-year actuarial local control rate of 89.3% compared with 59.0% and 8.1% for those treated with 36–53.9 Gy and less than 36 Gy. The optimal treatment dose has not been defined yet and should be the subject of future studies.

### Toxicity

In our study, treatment was well tolerated. There were no grade 4 events and only one case of grade 3 gastritis and epidermitis. All of the other events were grades 1 or 2. The most common acute toxicities were nausea and pain at the time of treatment. None of the toxicities prevented any patient from completing the treatment and were transitory. Rusthoven et al. have reported similar results with also one grade 3 event and no grade 4 events [17]. Lee et al. have reported two cases of grade 3 liver enzymes-related toxicities and six acute grade 3 toxicities in the form of two case of gastritis, two cases nausea, one lethargia, and one thrombocytopenia [18]. They also had one grade 4 toxicity in the form of thrombocytopenia. A longer follow-up period is necessary to detect potential long-term toxicities.

### SBRT and previous chemotherapy regimens

We wondered whether or not pre-treated patients with metastatic disease could benefit from SBRT. Most of our patients had received prior chemotherapy (91%), with a high number of chemotherapy regimens, 27% of them receiving more than three regimens, which may have increased the radioresistance of the metastases. We found that a patient’s history of prior chemotherapy was a major risk factor in recurrence outside the treatment volume (HR = 4.51, 95% CI: 1.10–18.47, p = 0.007). Also, the number of previous chemotherapy regimens administered or progression while receiving chemotherapy significantly correlates with a higher risk of failure. One hypothesis that could explain this finding could be that the previous chemotherapy regimens, received by the patients, selected tumoral clones with a lower sensitivity to radiation, even if no study has been published to prove it. This suggests that SBRT should perhaps be used as a local treatment for metastases before the administration of several systemic therapies.

## Conclusion

Ablative SBRT for liver and lung metastases achieves high 1- and 2-year local control rates, while minimal and invasive. Toxicitiy was very low consisting mainly of grade 1 and 2 nausea. The results indicate that ablative SBRT is a promising option even for pre-treated patients with oligometastases. Comparative studies are needed to better assess this issue.

## Competing interests

Radiation Therapy Department received a research grant from Accuray.

## Authors' contributions

IF, XM and EL conceived the study. IF collected data and drafted the manuscript .JEB, SD, HJ, BP, TL, XM and EL participated in coordination and helped to draft the manuscript. AK performed the statistical analyses. EL provided mentorship and edited the manuscript. All authors have read and approved the final manuscript.
